# The Neuropsychology of Male Adults With High-Functioning Autism or Asperger Syndrome†

**DOI:** 10.1002/aur.1394

**Published:** 2014-06-05

**Authors:** C Ellie Wilson, Francesca Happé, Sally J Wheelwright, Christine Ecker, Michael V Lombardo, Patrick Johnston, Eileen Daly, Clodagh M Murphy, Debbie Spain, Meng-Chuan Lai, Bhismadev Chakrabarti, Disa A Sauter, MRC AIMS Consortium, Simon Baron-Cohen, Declan G M Murphy

**Affiliations:** Sackler Institute for Translational Neurodevelopment, Institute of Psychiatry, King's College LondonLondon, United Kingdom; Autism Research Centre, Department of Psychiatry, University of CambridgeCambridge, United Kingdom; Cancer Sciences, University of SouthamptonSouthampton, United Kingdom; Department of Psychology, University of CyprusNicosia, Cyprus; Center for Applied Neuroscience, University of CyprusNicosia, Cyprus; Department of Psychiatry, College of Medicine, National Taiwan UniversityTaipei, Taiwan; School of Psychology and Clinical Language Sciences, University of ReadingBerkshire, United Kingdom; Department of Social Psychology, University of AmsterdamAmsterdam, the Netherlands; Autism Research Group, University of OxfordOxford, United Kingdom

**Keywords:** autism spectrum disorder, cognitive profiles, autistic symptomatology, comorbid psychopathology, support vector machine classification, autistic subtypes

## Abstract

Autism Spectrum Disorder (ASD) is diagnosed on the basis of behavioral symptoms, but cognitive abilities may also be useful in characterizing individuals with ASD. One hundred seventy-eight high-functioning male adults, half with ASD and half without, completed tasks assessing IQ, a broad range of cognitive skills, and autistic and comorbid symptomatology. The aims of the study were, first, to determine whether significant differences existed between cases and controls on cognitive tasks, and whether cognitive profiles, derived using a multivariate classification method with data from multiple cognitive tasks, could distinguish between the two groups. Second, to establish whether cognitive skill level was correlated with degree of autistic symptom severity, and third, whether cognitive skill level was correlated with degree of comorbid psychopathology. Fourth, cognitive characteristics of individuals with Asperger Syndrome (AS) and high-functioning autism (HFA) were compared. After controlling for IQ, ASD and control groups scored significantly differently on tasks of social cognition, motor performance, and executive function (*P*'s < 0.05). To investigate cognitive profiles, 12 variables were entered into a support vector machine (SVM), which achieved good classification accuracy (81%) at a level significantly better than chance (*P* < 0.0001). After correcting for multiple correlations, there were no significant associations between cognitive performance and severity of either autistic or comorbid symptomatology. There were no significant differences between AS and HFA groups on the cognitive tasks. Cognitive classification models could be a useful aid to the diagnostic process when used in conjunction with other data sources—including clinical history. ***Autism Res*** *2014, 7: 568–581.* © 2014 International Society for Autism Research, Wiley Periodicals, Inc.

## Introduction

The Medical Research Council-Autism Imaging Multicentre Study (MRC-AIMS) is a UK-based multidisciplinary collaborative project to study brain anatomy and connectivity in male adults with autism spectrum disorder (ASD). As well as neuroimaging, participants completed a series of diagnostic assessments, neuropsychological tests, and questionnaires. The latter measures were selected to ensure that the samples were well described and to provide behavioral correlates for the brain analyses. This paper describes the battery of neuropsychological tests and questionnaires and explores whether cognitive measures can reliably distinguish between ASD and control groups, or ASD subtypes, and how cognitive test performance relates to ASD symptom profile or associated psychiatric symptoms.

Research into cognitive differences in ASD has been driven by three highly influential theories: the “Theory of Mind” account [ToM; Baron-Cohen, Leslie, & Frith, 1985; Lombardo & Baron-Cohen, 2010 ] proposes that people with ASD have a reduced ability to attribute independent mental states to self and others to predict and explain actions; the theory of “executive dysfunction” [Ozonoff, Pennington, & Rogers, 1991; Rumsey & Hamburger, 1988 ] posits that ASD symptoms are a result of impairments in executive functions, including planning, inhibition, flexibility, and working memory; and finally, the “weak central coherence” theory [Frith, 1989; Happé & Frith, 2006 ] suggests that people with ASD have a cognitive style that favors processing of local, detailed information over global, holistic information. These three leading cognitive theories have undergone several modifications, and it is generally accepted that none of the theories can explain all cognitive and behavioral symptoms of ASD, but that each has the capacity to account for a wide range of atypical behaviors common to ASD [Happé & Ronald, 2008 ]. A vast amount of research on cognitive skills in people with ASD has been generated, albeit with conflicting results.

For example, deficits in social cognition have been reported in ToM [Baron-Cohen, O'Riordan, Stone, Jones, & Plaisted, 1999; Baron-Cohen, Wheelwright, Hill, Raste, & Plumb, 2001a; Castelli, Frith, Happé, & Frith, 2002; Happé, 1994 ], and in emotion recognition in high-functioning adults with ASD [Bal et al., 2010; Golan, Baron-Cohen, & Hill, 2006; Golan, Baron-Cohen, Hill, & Rutherford, 2007; Wallace et al., 2011; see Uljarevic & Hamilton, 2013 for a review]. However, other studies have reported that high-functioning adolescents and children with ASD perform as well as controls on ToM tasks [Scheeren, Koot, Mundy, Mous, & Begeer, 2013 ], and some tasks of emotion recognition report that adults with ASD are not necessarily impaired [Adolphs, Sears, & Piven, 2001; Rutherford & Towns, 2008 ]. Executive function deficits, including problems with generating ideas [Boucher, 1988; Low, Goddard, & Melser, 2009 ] and selective inhibitory impairments [Adams & Jarrold, 2012 ], have been reported in ASD. However, it has been suggested that poor performance on such tasks may reflect difficulties understanding experimenters' expectations rather than any specific deficit in executive function [White, 2013 ]. One study tested multiple components of executive function in 30 young ASD adults, and results were variable even within this participant group: they reported impairments in spatial working memory, but no impairments in planning, cognitive flexibility, and inhibition [Sachse et al., 2013 ]. With respect to studies of central coherence in ASD, many studies have reported that children and adults with ASD outperform typical controls on tasks where a local processing bias is advantageous [Bonnel et al., 2010; Jolliffe & Baron-Cohen, 1997; Shah & Frith, 1983, 1993 ], but others have not replicated this [Lai et al., 2012a; White & Saldana, 2011; for a review, see Happé & Frith, 2006 ].

These inconsistencies may partially be explained by small sample sizes or poor task selection [Charman et al., 2011 ], or by heterogeneity within the autism spectrum [Brock, 2011 ]. This heterogeneity is reflected in both the variation in type and severity of autistic symptoms, as well as in the differing degrees of comorbid psychopathology within the autistic spectrum. Therefore, if different studies sample differently from this heterogeneous group, conflicting findings might be predicted. In the present study, we aimed to address these limitations, and a number of others, in the following ways.

First, with respect to sample size, the current study includes 178 participants, which is sufficient to detect an effect size as small as Cohen's d = 0.3 at a power of 0.8. With the much smaller samples often used in studies of cognition in ASD, smaller effects may not reach significance and may not be reported.

Second, most studies have tested only a narrow selection of cognitive skills, making cross-task comparison difficult. The present study sampled a wide range of cognitive abilities including emotion recognition, theory of mind, specific executive functions, phonological memory, central coherence, and dexterity. In addition, it may be important to look at skills in combination since cognitive skills do not operate in isolation (e.g., verbal fluency is dependent on general processing speed, [Spek, Schatorjé, Scholte, & van Berckelaer-Onnes, 2009 ]; or some executive function tasks require a level of theory of mind, such as reflecting on one's own plans and goals, see White, 2013). Here, in addition to the traditional method of group comparisons on individual measures, we also used support vector machine (SVM) algorithms, a supervised multivariate classification method, which has proved useful, although not perfect, at distinguishing clinical groups using neuroimaging data [Ecker et al., 2010 ]. Here, SVM has been used with traditional neuropsychological data for the first time, and we aim to test whether multivariate pattern information could also be useful for distinguishing between two groups.

Third, most previous studies compare average performance of an ASD group with average performance of a non-ASD group. This approach ignores heterogeneity within the ASD sample, yet it is well established that the condition is a “spectrum” and that presentation varies enormously within the spectrum. Therefore, we examined whether scores on cognitive tests were correlated with overall symptom severity, and with severity of symptoms on separate domains. Some studies have examined this—e.g., deficits in executive function have been associated with social [Happé & Frith, 2006; Ozonoff et al., 1991 ] and nonsocial behaviors in ASD [Hill, 2004 ]. Brunsdon and Happé [2014 ] have recently reviewed the literature on the relationship between symptoms and neuropsychological/cognitive test performance in ASD groups, most of which has concerned children.

Fourth, ASD adults often have high levels of comorbid symptomatology, particularly depression, anxiety, and obsessionality [Joshi et al., 2013; Russell, Mataix-Cols, Anson, & Murphy, 2005 ], yet these factors are generally not considered in studies of cognition in ASD. Reports on cognitive ability in adults with depression, anxiety, and obsessive–compulsive disorder (OCD) are mixed [Castaneda, Tuulio-Henriksson, Marttunen, Suvisaari, & Lönnqvist, 2008 ], although one well-replicated finding is that executive function deficits are evident in individuals with depression [Fossati, Amar, Raoux, Ergis, & Allilaire, 1999; Marazziti, Consoli, Picchetti, Carlini, & Faravelli, 2010; Smith, Muir, & Blackwood, 2006 ], anxiety [Airaksinen, Larsson, Lundberg, & Forsell, 2004 ], and OCD [Cavallaro et al., 2003 ]. In the current study, we anticipated that ASD participants would have elevated levels of depression, anxiety, and obsessionality and examined the relationship between cognitive performance and degree of comorbid psychopathology. Significant associations could be useful in two respects: cognitive tasks could be used to predict the development of psychopathology, or differing levels of comorbid symptomatology could account for variation in cognitive ability.

Finally, we compared cognitive profiles of two ASD diagnostic subtypes—Asperger syndrome (AS) and high-functioning autism (HFA). A diagnostic distinction between these groups has, until now, been made on the basis of the presence of a language delay in HFA individuals and no delay in AS. Differences in linguistic ability have been reported in children with HFA and AS [Noterdaeme, Wriedt, & Hohne, 2010; Sahyoun, Soulieres, Belliveau, Mottron, & Mody, 2009 ], however review papers have concluded that the subtypes cannot be reliably distinguished on the basis of diagnostic criteria and cognitive profile [Macintosh & Dissanayake, 2004; Witwer & Lecavalier, 2008 ]. In line with this view, the fifth revision of the Diagnostic and Statistical Manual (DSM-5) collapses these diagnostic categories (along with Pervasive Developmental Disorder-Not Otherwise Specified) into a single category of ASD. Nevertheless, confirming whether any differences do exist is of interest because distinct cognitive profiles may be useful for clinical intervention and prognosis.

To summarize, the present study measured cognitive functioning and symptom profiles in a group of 178 male adults of normal intelligence, where half the participants were on the autism spectrum and half were neurotypical. We aimed to assess the utility of cognitive measures to predict diagnostic group membership, and to indicate severity of symptoms of ASD or commonly associated conditions.

## Method

### Participants

Eighty-nine male adults with ASD and eighty-nine matched neurotypical controls aged 18–43 years were recruited and assessed at one of the three AIMS-UK centers: the Institute of Psychiatry, London; the Autism Research Centre, University of Cambridge; the Autism Research Group, University of Oxford. All participants were right-handed. Approximately, equal ratios of cases to controls were recruited at each site: London, 41 ASD and 41 controls; Cambridge, 30 and 32; Oxford, 18 and 16, respectively.

Exclusion criteria for all participants included a history of major psychiatric disorder (with the exception of major depressive or anxiety disorders), head injury, genetic disorder associated with autism (e.g., fragile X syndrome, tuberous sclerosis), or any other medical condition affecting brain function (e.g., epilepsy). All ASD participants were diagnosed with ASD according to ICD-10 research criteria. If a language delay (no use of single words before 24 months, or no phrases before 33 months) was recorded on the Autism Diagnostic Interview-Revised [ADI-R; Lord, Rutter, & Couteur, 1994 ], HFA was diagnosed (*N* = 34). If no language delay was recorded on the ADI-R, AS was diagnosed (*N* = 55).

The study was given ethical approval by the National Research Ethics Committee, Suffolk, UK. All volunteers gave written informed consent.

### Measures

All participants completed a series of background measures assessing symptomatology and intelligence, and a series of neuropsychological tests.

#### Background measures

ASD diagnosis was confirmed using the ADI-R, which is a semi-structured interview conducted with parents or caregivers. It was allowed for participants to be 1 point below cutoff for one of the three ADI-R domains in the diagnostic algorithm. The Autism Diagnostic Observation Schedule Generic [ADOS-G; Lord et al., 2000 ], a semi-structured, standardized observational assessment, was used to assess current symptoms for all participants with ASD.

All participants completed three questionnaires assessing autistic traits (Autism Spectrum Quotient; AQ) [Baron-Cohen, Wheelwright, Skinner, Martin, & Clubley, 2001b ], empathy (Empathy Quotient; EQ) [Baron-Cohen & Wheelwright, 2004 ], and systemizing (Systemizing Quotient; SQ-R) [Wheelwright et al., 2006 ]. These instruments show association with common genetic polymorphisms [Chakrabarti et al., 2009 ] and are widely used both for screening for ASD and for measuring these traits dimensionally in the general population.

Participants also completed three questionnaires measuring symptoms of depression, anxiety, and obsession and compulsion. The Beck Depression Inventory [BDI; Beck, Steer, & Brown, 1996 ] and the Beck Anxiety Inventory [BAI; Beck & Steer, 1990 ] each includes 21 items and gives a maximum score of 63. The Obsessive–Compulsive Inventory-Revised [OCI-R; Abramowitz & Deacon, 2006 ] includes 18 items and gives a maximum score of 72.

The Wechsler Abbreviated Scale of Intelligence [WASI; Wechsler, 1999 ] was used to assess the general cognitive abilities of all participants. The WASI comprises four subtests, two verbal and two performance, and yields three standardized index scores: Verbal IQ (VIQ), Performance IQ (PIQ) and Full Scale IQ (FSIQ).

#### Neuropsychological tests

Tasks were selected to test the core domains considered abnormal in ASD based on the extant literature. Tests tapped emotion processing, theory of mind, language/phonological memory, executive functions, central coherence, and manual dexterity/handedness. For further details of tests, see supplementary materials.

1. **The Karolinska Directed Emotional Faces (KDEF): Emotion recognition** [Lundqvist, Flykt, & Öhman, 1998 ]. Participants indicated which emotion (happy, sad, angry, disgust, fear, surprise, or neutral) was displayed by a color face shown on a computer screen, using a 7-alternative forced choice task [Sucksmith, Allison, Baron-Cohen, Chakrabarti, & Hoekstra, 2013 ]. There were 140 trials. Dependent variables were percentage accuracy and mean reaction time.
2. **The “Reading the Mind in the Eyes” Task (RMET): Emotion recognition** [Baron-Cohen et al., 2001a ]. Participants were shown a black-and-white photograph of eyes and selected which word, from a choice of four, best described what the person in the photograph was thinking or feeling. There were 36 trials. Dependent variables were accuracy (total correct) and mean reaction time.
3. **The Frith-Happé Animations Test: Theory of mind** [Abell, Happe, & Frith, 2000 ]. Participants viewed six silent animations featuring two triangles interacting in such a way as to convey intentions toward the other character's mental state (coaxing, mocking, seducing, surprising; ToM animations) or physical state (leading, fighting; “goal-directed” animations). Participants responded verbally to the question “What happened in the cartoon?” Responses were scored for complexity of mental state terms used (“intentionality”; 0–3) and accuracy of answer given (“appropriateness”; 0–2). Summed scores for intentionality and for appropriateness were the variables calculated for ToM and goal-directed animations.
4. **Story Test: Theory of mind.** Participants were asked a read a story and answer second-order false belief and justification questions. Answers were scored 0 (don't know, incorrect), 1 (partially correct), or 2 (fully and explicitly correct), forming the dependent variable.
5. **FAS Task: Generativity.** Participants were asked to produce orally as many words as possible beginning with a particular letter (F, then A, then S). They had 60 sec per letter. The dependent variable was the number of words produced.
6. **Nonword Repetition (NWR): Phonological memory** [Adapted from Gathercole, Willis, Baddeley, & Emslie, 1994 ]. The participant heard a nonword aloud (e.g., “tirroge”) and then attempted to repeat it immediately. There were 28 trials. The dependent variable was the number of correct responses.
7. **Go/No Go Test: Attention/inhibition** (executive function), [Adapted from Rubia et al., 2001 ]. Participants were asked to indicate whether a series of arrows were pointing left (press “1”), right (press “2”), upward (no key press). There were 300 trials. Dependent variables were errors of omission (as % of trials), errors of commission (as % of trials) and beta, a summary measure indexed according to the signal detection theory [Green & Swets, 1966 ].
8. **Embedded Figures Test (EFT): Central coherence** [Witkin, Oltman, Raskin, & Karp, 1971 ]. Participants were asked to locate a nonmeaningful geometric figure (target) within a larger complex form. There were 12 items. The dependent variables were total correct and mean time to find the shape per trial (in seconds).
9. **Purdue Pegboard Test: Manual dexterity** [Tiffin & Asher, 1948 ]. In the first three subtests, subjects had 30 seconds to fill holes with pegs with the right hand (right hand) then the left hand (left hand), and finally with both hands (both hands) alternatively. Dependent variables were number of holes filled for each subtest and for the sum of the three subtests. In a fourth subtest, participants assembled a peg, then a washer, then a collar, then another washer, as many times as they could in 60 sec. This last dependent variable was the number of parts correctly assembled.

### Procedure

Before participants attended a testing center, they completed some information (date of birth, ethnicity and level of education, details of any regular medication) on a secure web site. They indicated whether they had ever been diagnosed with any of the following: ASD, attention deficit/hyperactivity disorder (or hyperkinetic disorder), OCD, Tourette's syndrome, language delay, epilepsy, depression, schizophrenia, bipolar disorder, personality disorder, fragile X syndrome, tuberous sclerosis, or general learning disability.

The questionnaires and tasks available for completion before the day of the appointment were (as they were named on the web site, with their usual name in brackets): Your Personality Questionnaire (AQ), Your Feelings Questionnaire (EQ), Your Interests Questionnaire (SQ-R), The Eyes Test (RMET), Go/No Go Test. Participants were reminded that they should complete all the questionnaires and tests by themselves. Participants without access to the internet completed these tasks during their appointment.

On the day of the appointment, the ASD participants first had an ADOS-G module-4 assessment and then completed the WASI. The control participants started the day with the WASI. The remaining tasks (Faces Test (KDEF), Animations Test, Story Test, FAS, non-word repetition (NWR), embedded figures test (EFT), and Purdue pegboard) were completed in a randomized order. While the ASD participants were being assessed, an ADI-R was carried out with a parent.

### Method of Analysis

#### Calculating the empathizing–systemizing discrepancy

E–S discrepancy, referred to as the “*D*-score” [Goldenfeld, Baron-Cohen, & Wheelwright, 2005; Lai et al., 2012b ] was quantified as the difference between standardized EQ and SQ-R scores. The EQ and SQ-R scores were standardized by subtracting the population mean from the raw score then dividing by the maximum possible score: S = (SQ-R-<SQ-R>)/150 and E = (EQ-<EQ>)/80, where <SQ-R> and <EQ> were the estimated population means (55.6 for SQ-R and 44.3 for EQ) derived from a previous large-scale UK study (*N* = 1761) [Wheelwright et al., 2006 ]. The discrepancy between systemizing and empathizing was then quantified as *D =* (S-E)/2. Larger *D*-scores indicate a stronger drive to systemize than to empathize, smaller *D*-scores indicate a stronger drive to empathize than to systemize.

#### Comparison of participant groups

The ASD and control groups were compared on all neuropsychological measures and questionnaire scores using *t*-tests. AS and HFA groups were then compared in the same way. Although some measures were not normally distributed, parametric tests were used because the sample sizes in the current study are considered large enough to be robust to deviations from normality [Skovland & Fenstad, 2001 ]. *P*-values were Bonferroni adjusted to correct for multiple comparisons, therefore a *P*-value of less than 0.002 was considered significant.

#### Classification using support vector machine (SVM)

A linear SVM was used to classify between individuals with ASD and controls, and between AS and HFA participants, on the basis of their task performance on a set of 12 variables. The 12 variables were VIQ, PIQ, and ten dependent variables from the neuropsychological/experimental tasks. Dependent variables for this analysis were selected so as not to be inherently interdependent; hence one variable was selected from each test (with the exception of the ToM animations, where intentionality and appropriateness scores are in principle orthogonal). Variables were chosen based on data distribution (e.g., no floor or ceiling effects) and/or conventional use in the research literature.

Classification using SVM has been described in detail elsewhere [Burges, 1998; Schoelkopf & Smola, 2002 ]. Briefly, SVM is a supervised multivariate classification method where input data are classified into two classes (e.g., individuals with ASD and neurotypicals) by identifying a separating hyperplane or decision boundary, which maximizes the margin (i.e., distance from the hyperplane to the closest data points). The algorithm is initially trained on a subset of the data to find a hyperplane that best separates the input space according to the class labels (e.g., − 1 for cases, + 1 for controls). This is achieved by maximizing the margin (i.e., distance from the hyperplane to the closest data points; [Vapnik, 1995 ]. Once the decision function is learned from the training set, it can be used to predict the class of a new set of test examples.

Our implementation used LIBSVM software (http://www.csie.ntu.edu.tw/∼cjlin/libsvm/) implemented in Matlab with a linear kernel and a regularization parameter (C) set to the default of 1. Because each variable in its raw form was not scaled similarly, we used a procedure to scale each variable between values of − 1 and 1. This reduced feature swamping effects of variables with large values and ranges compared to other variables. Scaling parameters were estimated on the training data within each fold of the cross validation loop and were then used to transform the test data. We trained and tested the classifier using a leave-two-out cross validation scheme, whereby on each cross validation fold, one individual from each group is left out as “test” cases, and the remaining individuals are used as the training set. To evaluate performance of the classifier, we used measures of accuracy, sensitivity, and specificity. Sensitivity and specificity are defined as:

sensitivity = TP/(TP + FN)

specificity = TN/(TN + FP) where TP is the number of true positives (i.e., the number of ASD individuals correctly classified), TN is the number of true negatives (i.e., number of neurotypical individuals correctly classified as controls), FP is the number of false positives (i.e., number of controls classified as ASD individuals), and FN is the number of false negatives (i.e., number of ASD individuals classified as controls). These performance metrics were also tested under conditions where the class labels (e.g., controls or ASD) were completely randomized (i.e., permutation test with 10,000 permutations) in order to evaluate the probability of getting specificity and sensitivity values higher than the ones obtained during the cross-validation procedure by chance.

The SVM analysis excludes participants with missing values. The remaining sample size was 58 ASD (35 AS, 23 HFA) and 66 controls.

#### Associations between cognitive measures and clinical symptoms

A correlation matrix was constructed to investigate associations between ASD symptom measures (ADI-R/ADOS-G/AQ/D-score) and cognitive measures, and between the comorbid symptom measures (BDI, BAI, OCI-R) and cognitive measures, for all the subjects together and for the ASD group and control group separately. Because it was predicted that scores on BDI, BAI, and OCI-R would be associated with executive function, we included all measures of the Go-No-Go (attention/inhibition) task in the correlation matrix. Where significant associations were found analyses of covariance (ANCOVAs) were conducted, using the symptom measure as a covariate, to establish whether differences on cognitive measures between cases and controls remained significant.

## Results

### Comparison of ASD and Control Groups

#### Participant characteristics (Table 1)

**Table 1:** Participant Characteristics: Mean (SD). ^SVM^Measures Indicate Those That Were Used in the Support Vector Machine (SVM)

	ASD	Control	Effect size of group difference ASD: Control	AS	HFA	Effect size of group difference AS:HFA
N	89	89		55	34	
Age	26 (7)	28 (6)	0.26	28 (7)	24 (6)	0.55*
VIQ^SVM^	110 (14)	109 (13)	0.05	113 (15)	106 (12)	0.46*
PIQ^SVM^	108 (16)	116 (12)	0.58**	109 (17)	106 (15)	0.19
FIQ	110 (15)	114 (12)	0.28	112 (15)	107 (13)	0.36
AQ	30 (9)	15 (6)	2.10**	30 (9)	30 (8)	0.06
*D*-score	0.15 (0.12)	0.02 (0.10)	1.26**	0.15 (0.12)	0.15 (0.11)	0.08
BDI	12.5 (10.2)	5.9 (5.7)	0.83***	13.8 (11.4)	10.4 (7.7)	0.36
BAI	12.0 (11.0)	4.9 (5.7)	0.85***	13.7 (11.9)	9.2 (8.7)	0.44
OCI-R	23.9 (14.5)	8.7 (6.8)	1.71***	24.9 (15.6)	22.1 (12.6)	0.20

*P < 0.05; **P < 0.01; ***P < 0.001.

VIQ, verbal IQ score; PIQ, performance IQ score; AQ, Autism Quotient score; *D*-score, empathizing–systemizing discrepancy.

The ASD group had a significantly lower PIQ than the controls. As expected, ASD scored significantly higher than the control group on all measures of symptomatology (AQ, *D*-score, BDI, BAI, and OCI-R).

#### Performance on cognitive tasks (Table 2)

**Table 2:** Neuropsychological Measures Comparing Control and ASD Performance: Mean (SD). ^SVM^Measures Indicate Those That Were Used in the Support Vector Machine (SVM)

Ability measured	Task measure	ASD mean (SD)	Control mean (SD)	ASD (*N*)	Control (*N*)	*P*-Value	Effect size (Cohen's d)	Covaried for PIQ (*P*-value)
1. Emotion recognition	KDEF—All emotions %^SVM^	83 (10)	85 (8)	82	84	0.25	0.20	1.0
	KDEF—All emotions RT	3062 (912)	2644 (583)	82	84	0.001*	0.55	< 0.001
2. Emotion recognition	Eyes RT	7009 (2469)	6401 (1776)	85	86	0.07	0.28	0.07
	Eyes Correct^SVM^	22 (6)	27 (4)	85	86	< 0.001*	0.98	< 0.001
3. Theory of mind—Animations task	GD Intentionality	4.2 (1.1)	4.5 (0.7)	64	71	0.07	0.33	0.11
	GD Appropriateness	2.7 (1.1)	3.0 (0.9)	64	71	0.05	0.30	0.20
	TOM Intentionality^SVM^	9.3 (1.8)	10.3 (1.6)	64	71	0.001*	0.59	0.01
	TOM Appropriateness^SVM^	3.1 (2.1)	4.8 (1.9)	64	71	< 0.001*	0.85	< 0.001
4. ToM	Story Test^SVM^	1.3 (1.2)	1.2 (1.1)	89	89	0.55	0.09	0.83
5. Generativity	FAS^SVM^	39.0 (13.3)	43.1 (12.5)	87	88	0.04	0.32	0.34
6. Phonological memory	Nonword Repetition^SVM^	21.3 (4.6)	22.8 (3.5)	85	86	0.02	0.37	0.05
7. Attention/Inhibition	Beta^SVM^	1.1 (0.8)	1.4 (1.1)	87	81	0.03	0.31	0.04
	Omission %	2.3 (2.9)	0.8 (1.7)	84	82	< 0.001*	0.63	< 0.001
	Commission %	4.9 (4.0)	2.4 (3.0)	84	82	< 0.001*	0.70	0.01
8. Central coherence	EFT Score	9.0 (3.0)	9.7 (2.2)	82	87	0.08	0.27	0.23
	EFT RT^SVM^	16.3 (7.9)	14.9 (7.6)	82	87	0.25	0.18	0.55
9. Manual dexterity	Peg RH	12.9 (2.4)	14.3 (2.0)	87	88	< 0.001*	0.63	< 0.001
	Peg LH	12.2 (2.5)	13.6 (2.0)	87	88	< 0.001*	0.61	< 0.001
	Peg both	11.1 (4.2)	13.0 (2.7)	87	88	0.04	0.53	0.27
	Peg assembly^SVM^	28.4 (8.4)	34.5 (8.1)	87	88	< 0.001*	0.74	< 0.001

* To correct for multiple comparisons: *P* < 0.002 is considered significant.

Of the nine neuropsychological tests, significant differences between ASD and control groups were found on five after controlling for PIQ, although not every variable from these tasks showed a significant effect of group. These were the KDEF task and eyes task (emotion recognition), animations task (ToM), the Go/No-Go task (executive function: attention and inhibition), and pegboard (manual dexterity).

**Figure 1:**
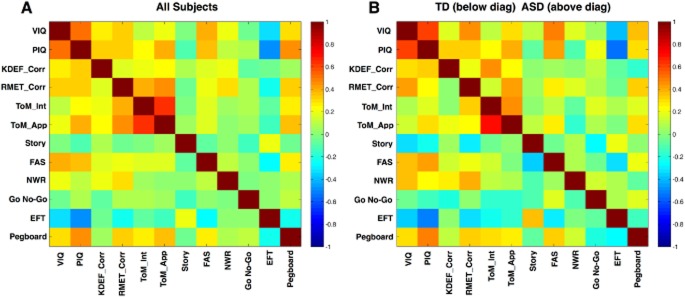
Correlation matrices for all variables entered into the SVM. Panel A shows the correlation matrix for all subjects. Panel B shows the correlation matrix for separate groups (TD subjects below the diagonal and ASD subjects above the diagonal).

Correlations between scores on the 12 tasks used in the SVM were also conducted across the whole group and in cases and controls separately (Fig. 1). The tests correlated with other to varying degrees, with *P*-values ranging from < 0.01 to .6.

#### Group classification using the support vector machine (SVM)

Overall, the set of 12 variables achieved good classification accuracy (81%), sensitivity (78%), and specificity (85%), and each of these were highly significant compared to chance simulations using permutation tests (accuracy *P* = 9.99 × 10^−5^; sensitivity *P* = 9.99 × 10^−5^; specificity *P* = 6.99 × 10^−4^). Thus, the probability of obtaining such performance metrics under conditions that assume no true difference between the groups is extremely low.

#### Associations between cognitive measures and ASD symptoms (Table 3)

**Table 3:** Correlations Between ASD Clinical Symptom Measures and the 12 IQ/Neuropsychological Variables Used in the SVM in the ASD Group (Pearson's R-Value)

Measure	ADI-R total (social + communication + RRBI)	ADOS-G total (social + communication)	ADI-R social	ADI-R communication	ADI-R RRBI	ADOS-Social	ADOS-Communication
Verbal IQ^SVM^	0.01	−0.03	−0.05	0.00	0.17	−0.07	0.03
Performance IQ^SVM^	−0.03	−0.02	−0.09	−0.01	0.11	−0.03	0.01
All Emotions %^SVM^	0.11	−0.19	0.01	0.15	0.24*	0.10	0.03
Eyes Correct^SVM^	−0.12	−0.3	−0.18	−0.05	−0.01	−0.01	−0.05
ToM Intentionality^SVM^	−0.5	**−**0.26*	−0.03	0.03	−0.16	−0.24	−0.22
ToM Appropriateness^SVM^	0.01	**−**0.25*	−0.03	0.13	−0.14	−0.25	−0.22
Story Test^SVM^	0.19	0.07	0.17	0.21	−0.03	0.03	0.10
FAS^SVM^	0.09	−0.07	0.04	0.05	0.17	−0.08	−0.06
Nonword Repetition^SVM^	−0.16	0.23*	−0.12	−0.18	−0.02	0.26*	0.22*
Beta^SVM^	0.19	0.10	0.24*	0.26*	0.04	0.02	0.05
EFT RT^SVM^	0.04	0.06	−0.04	0.01	−0.07	−0.01	−0.11
Peg Assembly^SVM^	−0.02	−0.18	−0.09	−0.03	0.18	−0.14	−0.17

* Signifies *P* < 0.05.

After correcting for multiple correlations, no associations reached significance.

#### Associations between cognitive measures and comorbid psychopathology (Table 4)

**Table 4:** Correlations Between Questionnaires Measuring Clinical Symptoms and the 12 IQ/Neuropsychological Variables Used in the SVM in the ASD Group (Pearson's R-Value)

Measure	AQ *z*-core	*D*-score	BDI	BAI	OCI
Verbal IQ^SVM^	0.13	0.17	−0.06	−0.11	−0.75
Performance IQ^SVM^	0.17	0.27*	0.04	0.01	−0.06
All emotions %^SVM^	−0.13	0.03	−0.20	−0.11	−0.08
Eyes correct^SVM^	−0.10	−0.06	−0.06	−0.02	−0.03
TOM intentionality^SVM^	−0.15	−0.10	−0.03	−0.03	−0.10
TOM Appropriateness^SVM^	0.01	0.06	−0.27*	−0.22	−0.20
Story Test^SVM^	−0.07	0.01	−0.26*	−0.20	−0.09
FAS ^SVM^	0.08	0.11	0.14	0.15	0.10
Nonword repetition^SVM^	0.03	0.01	−0.17	−0.13	−0.14
Beta^SVM^	0.04	−0.01	−0.02	−0.02	−0.09
EFT RT^SVM^	−0.11	−0.09	−0.09	−0.06	−0.02
Peg assembly^SVM^	0.10	0.25*	0.08	0.14	0.01

* Signifies *P* < 0.05.

*D*-score: empathizing–systemizing discrepancy.

The BAI and OCI-R were not significantly associated with any cognitive variables. The BDI correlated significantly negatively with performance on the ToM Appropriateness task and on the Story Test, although the significance level did not survive Bonferroni corrections. Nevertheless, to establish whether the significant group difference that was found on the TOM appropriateness task was accounted for by level of depression, an ANCOVA was conducted between group (ASD, control) and scores on ToM appropriateness, covarying for BDI score. Group differences on ToM Appropriateness remained highly significant, F (1,130) = 10.63, *P* = 0.001.

Correlations were also run for the control group between the 12 task variables and symptomatology according to the AQ, *D*-scores, BDI, BAI, and OCI-R; no correlations were significant after Bonferroni corrections (all *P*'s > 0.02).

### Comparison of AS and HFA Groups

#### Participant characteristics (Table 1)

The AS group were significantly older and had higher VIQ than the HFA group, but the groups were closely matched on questionnaires measuring clinical symptoms (AQ, *D*-score, BDI, BAI, and OCI-R). There were no significant differences found on the ADOS-G (*P*'s > 0.05), however the HFA group scored higher (indicating greater impairment) than the AS group on the ADI-R on both the Social (*t*(87) = 2.75, *P* < 0.01; Cohen's d = 0.60) and Communication (*t*(87) = 2.54, *P* < 0.05; Cohen's d = 0.54) domains, although not on the Repetitive Behaviors and Restricted Interests domain (*P* > 0.5).

#### Performance on cognitive tasks (Table 5)

**Table 5:** Neuropsychological Measures Comparing AS and HFA Performance: Mean (SD). ^SVM^Measures Indicate Those That Were Used in the Support Vector Machine (SVM)

Task number. Ability measured	Measure	AS	HFA	AS (*N*)	HFA (*N*)	*P*-value	Effect size (Cohen's d)	Covaried for VIQ and age (*P*-value)
1. Emotion recognition	KDEF—All emotions % ^SVM^	83.2 (9.8)	83.2 (10.9)	51	31	0.68	0.00	0.43
	KDEF—All emotions RT	3079.0 (826.7)	3034.9 (1051.3)	51	31	0.83	0.04	0.35
2. Emotion recognition	Eyes RT	7120.5 (2644.1)	6816.2 (2157.6)	51	31	0.59	0.13	0.67
	Eyes correct ^SVM^	22.4 (6.0)	21.2 (5.2)	54	31	0.35	0.21	0.50
3. Theory of mind (animations task)	GD intentionality	3.9 (1.2)	4.5 (0.9)	38	26	0.04	0.55	0.05
	GD appropriateness	2.6 (1.2)	2.9 (1.0)	38	26	0.29	0.27	0.79
	ToM intentionality^SVM^	9.1 (2.0)	9.7 (1.3)	38	26	0.13	0.36	0.22
	ToM appropriateness^SVM^	2.5 (1.8)	3.9 (2.2)	38	26	0.01	0.67	0.01
4. ToM	Story test^SVM^	1.2 (1.2)	1.4 (1.2)	55	34	0.60	0.11	0.70
5. Generativity	FAS^SVM^	41.89 (12.9)	34.3 (12.6)	54	33	0.01	0.58	0.15
6. Phonological memory	Nonword repetition^SVM^	22.1 (3.8)	20.1 (5.6)	53	32	0.05	0.43	0.12
7. Attention/Inhibition	Beta^SVM^	1.2 (0.8)	1.0 (0.7)	52	32	0.35	0.27	0.26
	Omission %	2.3 (3.0)	2.3 (2.9)	52	32	0.96	0.00	0.70
	Commission %	4.8 (3.5)	5.0 (4.7)	52	32	0.83	0.04	0.97
8. Central coherence	EFT Score	9.0 (3.0)	9.0 (3.1)	54	32	0.99	0.00	0.86
	EFT RT^SVM^	16.4 (8.8)	16.0 (6.2)	51	31	0.80	0.06	0.47
9. Manual dexterity	Peg RH	12.9 (2.4)	12.9 (2.4)	54	33	0.91	0.02	0.96
	Peg LH	12.7 (2.3)	11.3 (2.7)	54	33	0.01	0.56	0.07
	Peg both	11.7 (3.8)	12.2 (4.9)	54	33	0.57	0.12	0.20
	Peg assembly^svm^	28.8 (8.8)	27.8 (7.7)	54	33	0.57	0.12	0.49

*To correct for multiple comparisons: *P* < 0.002 is considered significant (No values reached significance).

When age and VIQ were partialled out, and *P*-values were adjusted for multiple comparisons, there were no significant differences in performance on any of the cognitive tasks.

#### Diagnostic subtype classification using the SVM

When trying to separate individuals with AS from individuals with HFA, the classifier performed at 58% accuracy, 39% sensitivity, and 71% specificity and none were better than chance performance in permutation tests (accuracy *P* = 0.29; sensitivity *P* = 0.21, specificity *P* = 0.59).

## Discussion

The cognitive function of adults on the autistic spectrum has undergone extensive investigation, but the results of previous studies have been inconsistent. This may be partially due to methodology (small sample sizes, poor task selection), or due to heterogeneity within the autistic spectrum. In the current study, we attempted to address some of the limitations of previous studies and tested a large sample of male adults on a range of cognitive tasks. Half of the participants were on the autistic spectrum and half were not. We had four aims: first, to determine whether reliable group differences existed on performance on individual cognitive tasks or on a combination of tasks between cases and controls, and therefore whether these might be useful in categorizing individuals; second, to establish whether performance on the tasks was correlated with degree of autistic symptom severity within diagnostic groups, and third, with degree of comorbid psychopathology. Last, we examined whether cognitive profile distinguished putative subgroups within the autism spectrum.

The use of multiple neuropsychological tasks was justified since the correlation matrix demonstrated that different tasks were associated with one another to varying degrees, thus likely tapping different cognitive domains. Our results suggest that some of these tasks distinguished an ASD group from a neurotypical group (with comparable IQ). The control group significantly outperformed the ASD group on tasks tapping social cognition (KDEF, Eyes Test, ToM animations), executive function (Go No-Go task), and motor performance (pegboard), even when Performance IQ, which differed significantly between ASD and control groups, was partialled out. These highly significant results suggest that there are certain cognitive deficits that are characteristic of male adults on the autistic spectrum. However, there was no clear deficit on tasks tapping generativity (FAS task), phonological memory (nonword repetition), or central coherence (EFT task).

We also investigated whether a cognitive profile across a combination of tasks could distinguish between individuals in the ASD and control groups. ASD is a complex and heterogeneous condition; therefore, it is unlikely that any single model will classify cases and controls 100% accurately when compared to the outcome of gold-standard diagnostic measures (i.e., ADI-R and ADOS-G). Nevertheless, results of the SVM analysis indicated that participants could be accurately classified as ASD or control at a level that was much better than chance (78% sensitivity and 85% specificity). SVM from the same sample using magnetic resonance imaging (MRI) data from a 30-min structural scan allowed group distinction to be achieved at similar rate [90% sensitivity and 80% specificity; Ecker et al., 2010 ]. We suggest that using data sets from multiple models in conjunction (e.g., cognitive and MRI data), where each performs significantly better than chance, could provide valuable objective tools to aid the diagnostic process. This needs to be tested in “real-world” clinical situations where the comparison groups include people with other neurodevelopmental disorders, and/or those with complex personality structures seeking diagnosis relatively late in life.

Regarding our second aim, nonsignificant correlations indicated that ASD symptom severity was unrelated to the cognitive factors examined here. This highlights how variable the autistic spectrum can be, since an individual's symptom severity does not predict their skill level on any particular cognitive domain, and likewise, cognitive skill level is not indicative of ASD symptom severity. This supports the idea that underlying neuropsychological mechanisms may be the same even when clinical presentation is different, which has implications for clinical practice and genetic research.

As expected, the ASD group had elevated severity of comorbid psychopathology. However, the predicted association between measures of executive function and degree of depression, anxiety, or obsessionality was not significant. There were moderate associations indicating that increased depressive symptomatology was associated with poorer ToM, which is in line with previous reports of poor ToM in adults with depression [Fossati et al., 1999; Smith et al., 2006 ]. We suspected that elevated psychopathological symptoms in the ASD group might account for the poorer performance on the cognitive tasks when compared to the controls. However, the results did not support this, suggesting that the deficit in ToM was a factor of being on the autistic spectrum, not a factor of having comorbid symptoms of depression.

With respect to ASD diagnostic subtypes, there were no significant differences between AS and HFA groups on individual cognitive measures, and the multivariate SVM technique did not classify groups any better than chance. In terms of autistic symptom severity, we did find that the HFA group exhibited greater symptom severity than in the AS group in childhood (as measured by the ADI-R), but that symptom severity had leveled out by adulthood (as measured by the ADOS-G). This suggests that a language delay, which distinguishes HFA from AS, is associated with greater severity and wider range of autistic symptoms in childhood, but that these differences do not persist into adulthood with respect to behavioral or cognitive profiles. On balance, therefore, our data are consistent with the idea of collapsing subtypes within the autism spectrum in DSM-5.

## Limitations

The large battery of tasks may have led some participants to become tired, perhaps affecting performance. While we have incorporated a range of tests assessing the cognitive functions most commonly associated with ASD, we have not, of course, tested every possible cognitive function. Also, some cognitive functions were tested with multiple tasks (e.g., social cognition), but others were tested by only one task. A different set of tests might well have yielded different results. In addition, it would have been useful to include symptom measures of other commonly associated difficulties, notably ADHD. With regards to the sample, we had fairly large and well-defined ASD and control groups, but our results cannot be generalized to ASD individuals that are not in the average intelligence range, or to females with ASD [Lai et al., 2011, 2012a ]. There were also significant age differences between the HFA and AS groups. Last, our controls group comprised very healthy individuals without any other neurodevelopmental or mental health problems. Hence, we cannot state that our findings are specific to ASD.

## Conclusion

Male adults with ASD can be distinguished from those without ASD on the basis of their performance across a range of neuropsychological tasks. Performance on cognitive tasks in the present ASD sample could not be explained by the presence of additional psychopathology (e.g., anxiety, depression)—but neither could these additional symptoms be well predicted by the present cognitive tasks. Diagnostic subtypes within the autism spectrum do not seem to be distinguished by such tasks, once differences in IQ are controlled for. In sum, neuropsychological tasks may add information of use to clinicians assessing intellectually able adults with ASD, and, when used in conjunction with other data sources (e.g., clinical history and neuroimaging data), could provide valuable tools to assist diagnostic assessments and guide treatment.

## Supporting Information

**Appendix S1.** Details of Neuropsychological Tests
