# Supplementary material for: The Neuropsychology of Male Adults With High-Functioning Autism or Asperger Syndrome
Source: Autism Res. 2014 Jun 5;7(5):568–81. doi: 10.1002/aur.1394 (PMC4489335; doi:10.1002/aur.1394)
Supplement: Supplementary file 1 — Appendix S1. Details of Neuropsychological Tests [file aur0007-0568-sd1.doc]

Supplementary Material

Details of Neuropsychological Tests

***1. The Karolinska Directed Emotional Faces (KDEF; Lundqvist et al., 1998): Emotion recognition.*** The KDEF test comprises 140 natural faces shown in colour, with an equal number of male and female actors. There are 20 faces expressing each of the following emotions: happy, sad, angry, disgust, fear, surprise and neutral. Each face is presented in the middle of the screen with the emotion words underneath, numbered 1-7 to correspond with the response keys. Participants are asked to decide which emotion best described what the person was feeling, and made their response using the keys 1 to 7 (Sucksmith et al., 2012). The next trial appears once a participant has made their response, or after 20 seconds of time has lapsed. Dependent variables were accuracy and reaction times for each trial

***2. The Reading the Mind in the Eyes Task: Emotion recognition (Baron-Cohen et al., 2001).*** The online version of the Reading the Mind in the Eyes Task (RMET) comprises the same 36 items as are presented in the published version. Thirty-six black-and-white photographs of eyes are presented in a random order in the centre of the screen. Participants are asked to choose the word, from a choice of four, that best describes what the person in the picture is thinking or feeling. The four possible responses are shown at four corners surrounding the picture and are labeled with the response key in a manner that corresponds to the response keys on the keyboard. The next trial appears once a participant has made their response, or after 20 seconds of time has lapsed. Dependent variables were accuracy (total correct) and mean reaction time.

***3. The Frith-Happé Animations Test: Theory of Mind (Abell et al., 2000).*** This task assesses intuitive Theory of Mind or mentalising using silent animated geometric shapes moving about the screen. Six silent animations lasting 39-42 seconds each were shown on a computer screen. All of the animations featured a large red triangle and a small blue triangle moving about on a framed white background. The triangles move as if self propelled in each sequence. The animations convey different types of action / interaction through their kinetic patterns.

Four Theory of Mind (ToM) and two Goal Directed (GD) animations were displayed in a (pseudo) random order to each subject. The Theory of Mind animations were scripted as Coaxing, Mocking, Seducing and Surprising. They involved one character reacting to the other character’s mental state. In one animation the large triangle is coaxing the small triangle out of an enclosure, a second showed the small triangle mocking the larger triangle; another showed the small triangle seducing and persuading the other triangle to let it go free; a fourth sequence showed the small triangle hiding and surprising the other triangle. The Goal Directed actions included Leading and Fighting. The sequences involved one character responding to the other character’s behaviour and were therefore likely to evoke direct descriptions of interaction. One GD animation showed one triangle leading the other triangle and the second animation showed the triangles fighting with each other. In each sequence there was reciprocal interaction without the implication that one character was reacting to the others mental state.

The animations were presented on computer. To familiarise the participants with the task a practice Goal Directed animation was shown. After each animation the same question was asked “What happened in the cartoon?” The verbal responses were noted and while the participants were encouraged, they were not given feedback. The verbal responses were coded with respect to “intentionality” which means the degree of mental state attribution (0-3, with absence of mental state language at one pole and elaborate use of mental state language at the other) and “appropriateness” (0-2 with incorrect at one extreme and highly appropriate at the other). Summed scores for intentionality and for accuracy were the variables calculated for ToM and Goal-directed animations.

***4. Story Test: Theory of Mind.*** This was an online. Participants were asked a read a story and type an answer in response to 11 second-order false belief and justification questions. Answers were scored 0 (don’t know, incorrect), 1 (partially correct), or 2 (fully and explicitly correct), forming the dependent variable.

***5. FAS Task: Generativity.*** This task provides a measure of verbal fluency. Over three 60 second trials, participants were asked to produce orally as many words as possible, beginning with a specific letter (F, A or S). A score for each participant was calculated by summing each unique word produced, discounting proper nouns and words with the same stem but different endings (e.g. build, builds, building). The dependent variable was the number of words produced.

***6. Non-word Repetition: Phonological Memory (adapted from Gathercole et al., 1994).*** The Non-word Repetition test (NWR) was developed from the Children’s Test of Non-word Repetition CNRep. This is a test of phonological working memory. A non-word is an unfamiliar phonological form which can be read, written and repeated but has no conceptual meaning or semantic worth within the lexicon of the language. The participant hears such a non-word aloud (for example “tirroge”) and then attempts to repeat it immediately. The test consists of 28 non-words of different length (2, 3, 4, and 5 syllables) presented in the same order to all participants. The dependent variable was the number of correct responses.

***7. Go/No Go Test: Attention / Inhibition (Executive Function), (adapted from Rubia et al., 2001).*** Participants were told to expect a series of arrows, which could point to the left, right or up. They were asked to press the “1” key in response to left arrows, the “2” key in response to right arrows and not to press anything if the arrow pointed upwards. There were 300 trials, 110 trials each of left and right arrows (go trials) and 80 up arrows (no go trials). For each participant, trials were presented in the same fixed quasi-randomised order so that two ‘no go’ trials never occurred consecutively. Each trial was a maximum of 1200ms long, with a 100ms white screen between trials. As soon as the participant responded, the next trial began. Dependent variables were errors of omission (as % of trials), errors of commission (as % of trials) and beta, a summary measure indexed according to the signal detection theory (Green & Swets, 1966). Beta is calculated using the following equation:

ln(beta) = sensitivity d’ x criterion C.

***8. Embedded Figures Test (EFT): Central Coherence (Witkin et al., 1979).*** The EFT is a visual search and local processing task that involves locating a simple figure (target) within a larger complex form that has been designed to obscure or embed the simple figure. In the adult form of the EFT, the complex form is a non-meaningful geometric design.

Twelve items were administered in total. A maximum time of one minute was allowed for each item. The test was discontinued after four consecutive failures. The dependent variables were total correct and mean time to find the shape per trial (in seconds).

***9. Purdue Pegboard Test: Manual Dexterity (Tiffin & Asher, 1948).*** The Purdue Pegboard Test provides a measure fine motor hand function. The Purdue Pegboard (Model 32020; Lafayette Instrument Co., Lafayette, Indiana) is comprised of 50 holes arranged in two parallel columns and four cups located at the top of the board which contain pegs, washers and collars. There are four subtests. In each of the first three subtests, subjects have 30 seconds to fill the holes with pegs initially with the right hand (Right Hand) then with the left hand (Left Hand), and finally with both hands (Both Hands) simultaneously. The number of pins inserted for each subtest is counted and recorded. The sum of the test scores of Right Hand, Left Hand and Both was also calculated and recorded, forming the dependent variables.

In the last subtest, subjects had 1 minute to assemble in sequence a peg, a washer, a collar, and another washer with alternating hands starting with the right hand. The dependent variable was the number of parts assembled was counted and recorded (Assembly score). Since there were four parts in each assembly, if the subject made six complete assemblies for example, the score was 6 multiplied by 4 and recorded as 24. If there were incomplete assemblies correctly placed at the end of the minute, they were also added to the Assembly score.
